# Prevalence and risk factors of schistosomiasis and hookworm infection in seasonal transmission settings in northern Côte d’Ivoire: A cross-sectional study

**DOI:** 10.1371/journal.pntd.0011487

**Published:** 2023-07-17

**Authors:** Jules N. Kouadio, Jennifer Giovanoli Evack, Jean-Baptiste K. Sékré, Louise Y. Achi, Mamadou Ouattara, Jan Hattendorf, Oliver Balmer, Bassirou Bonfoh, Jakob Zinsstag, Jürg Utzinger, Eliézer K. N’Goran

**Affiliations:** 1 Unité de Formation et de Recherche Biosciences, Université Félix Houphouët-Boigny, Abidjan, Côte d’Ivoire; 2 Centre Suisse de Recherches Scientifiques en Côte d’Ivoire, Abidjan, Côte d’Ivoire; 3 Swiss Tropical and Public Health Institute, Allschwil, Switzerland; 4 University of Basel, Basel, Switzerland; 5 Epidemiology, Biostatistics and Prevention Institute, University of Zurich, Zurich, Switzerland; 6 Ecole de Spécialisation en Elevage et des Métiers de la Viande de Bingerville, Bingerville, Côte d’Ivoire; 7 Laboratoire National d’Appui au Développement Agricole, Abidjan, Côte d’Ivoire; Seoul National University College of Medicine, REPUBLIC OF KOREA

## Abstract

**Background:**

Schistosomiasis and hookworm infection remain public health problems in large parts of sub-Saharan Africa. The epidemiology of schistosomiasis and hookworm was studied in seasonal transmission settings in the northern part of Côte d’Ivoire.

**Methodology:**

In August 2018, a cross-sectional study was conducted. Urine and stool samples were collected from 742 individuals aged 6–96 years in 16 localities from four departments in northern Côte d’Ivoire. Urine samples were examined by a filtration method for quantification of *Schistosoma haematobium* eggs. Stool samples were subjected to duplicate Kato-Katz thick smears and eggs of *Schistosoma mansoni* and soil-transmitted helminths (STHs) were counted. Additionally, a questionnaire was administered to determine demographic characteristics and to identify risk factors of schistosomiasis and STHs. Malacologic surveys were carried out at water points that are contacted by humans and animals.

**Principal findings:**

The prevalence of schistosomiasis was very low. Only two cases of *S*. *mansoni* were found (0.3%, 95% confidence interval [CI]: 0.1–1.0%). The distribution of *S*. *haematobium* was focal, with cases found only in two departments; Ferkessédougou (5.4%, 95% CI: 2.5–9.9%) and Ouangolodougou (2.7%, 95% CI: 0.9–6.3%). Hookworm was the only STH species observed with a prevalence of 1.5% (95% CI: 0.8–2.8%). A higher risk of *S*. *haematobium* infection was observed in males compared to females, but the difference was not statistically significant (2.3% versus 1.3%, odds ratio [OR]: 1.5, 95% CI: 0.8–2.7). Participants aged 16–20 years showed the highest prevalence of *S*. *haematobium*. A total of 111 human- and animal-water contact points were identified at 47 water sources. Three potential intermediate host snails of schistosomes were collected; namely, *Bulinus forskalii* (n = 761), *Bulinus truncatus* (n = 205), and *Biomphalaria pfeifferi* (n = 1). Yet, only one specimen of *Bu*. *truncatus* was found to be shedding schistosome cercariae.

**Conclusions/Significance:**

This study confirms very low transmission of schistosomiasis and hookworm in northern Côte d’Ivoire. The establishment and rigorous implementation of integrated surveillance-response systems could lead to the elimination of schistosomiasis and hookworm in this part of Côte d’Ivoire.

## Author summary

Schistosomiasis and soil-transmitted helminthiasis, the latter including, hookworms, roundworms, and whipworms, are the most widespread parasitic neglected tropical diseases. As is the case in many sub-Saharan African countries, schistosomiasis and soil-transmitted helminthiasis are endemic in Côte d’Ivoire. Mass drug administration is the mainstay of the global control strategy against schistosomiasis and soil-transmitted helminthiasis and has been implemented in Côte d’Ivoire since 2013. The new road map put forward by the World Health Organization (WHO) for the control of neglected tropical diseases aims to eliminate schistosomiasis and soil-transmitted helminthiasis as a public health problem by 2030. In light of WHO’s goals, this study updates prevalence data for schistosomiasis and soil-transmitted helminthiasis and provides a deeper understanding of risk factors that drive transmission in the northern part of Côte d’Ivoire. The findings showed very low prevalence and transmission of schistosomiasis and hookworm. Participants aged 16–20 years and fishermen had the highest prevalence of urogenital schistosomiasis, with both males (2.3%) and females (1.3%) at risk of infection. The results corroborate findings from other investigations in sub-Saharan Africa, which confirm that current efforts to control schistosomiasis and soil-transmitted helminthiasis are successful. Nevertheless, further investment is still needed to reach elimination. In particular, a surveillance-response system would be a valuable tool in a low-prevalence context such as northern Côte d’Ivoire.

## Introduction

Schistosomiasis is a chronic parasitic disease caused by infection with trematode worms of the genus *Schistosoma* [1]. Schistosomiasis has a complex life cycle, involving freshwater snails as intermediate host, and human and other mammals as definitive hosts. Schistosomiasis remains a public health problem in tropical and subtropical regions of Africa, Asia, the Caribbean, and South America [2]. An estimated 779 million people are at risk of acquiring schistosomiasis [2], with some 241 million people requiring preventive chemotherapy in 2020 [3]. In 2018, the estimated global burden of schistosomiasis was 1.4 million disability-adjusted life years (DALYs) [4]. More than 90% of schistosomiasis cases are concentrated in sub-Saharan Africa, where the most relevant human-pathogenic species are *Schistosoma mansoni* and *S*. *haematobium*. As is the case with many neglected tropical diseases (NTDs), schistosomiasis primarily plagues marginalized communities, where there is no or only limited access to clean water and improved sanitation [5]. Following a World Health Assembly (WHA) resolution put forward in 2001, considerable resources have been dedicated to prevent and control schistosomiasis in humans, mainly by means of morbidity control through preventive chemotherapy with praziquantel. Over the past 15 years, schistosomiasis has decreased significantly in many countries in sub-Saharan Africa [6]. In order to contribute to the attainment of Sustainable Development Goal (SDG) 3, that is to ensure healthy lives and promote well-being for all at all ages, the new WHO road map targets the elimination of schistosomiasis and soil-transmitted helminthiasis (STH) as a public health problem by 2030, and the interruption of schistosome transmission in humans in selected countries by 2030 [7]. STH is caused by parasitic worm infections with three main species, namely *Ascaris lumbricoides*, hookworm, and *Trichuris trichiura* [8,9]. In 2018, the global burden of STH was estimated at 1.9 million DALYs [4]. Schistosomiasis also affects the livestock sector and hybrids between livestock and human schistosome species have been reported in humans [10–12].

In Côte d’Ivoire, schistosomiasis is endemic and caused by infection with *S*. *mansoni* and *S*. *haematobium* [13,14], with *Biomphalaria* and *Bulinus* serving as intermediate host snails, respectively. In 2013, the national prevalence of *S*. *mansoni* in school-age children was 37.7% based on an analysis of historical data [15]. Over the past decade, a preventive chemotherapy program was implemented, mainly targeting school-age children [16]. Consequently, the prevalence of schistosomiasis has declined, also in the northern part of the country, where transmission is seasonal [17]. Of note, livestock schistosomiasis is endemic in Côte d’Ivoire with high prevalences observed in the North [18]. In light of the new WHO road map, it is important to update the distribution of schistosomiasis and STH and deepen the understanding of risk factors that drive transmission.

This study aimed to assess the epidemiologic characteristics of schistosomiasis and STH in northern Côte d’Ivoire. The specific objectives were to determine the prevalence, distribution, and risk factors of schistosomiasis and STH. Particular emphasis was placed on freshwater snails in human- and animal-water contact sites. The study was part of a larger One Health project investigating transmission dynamics of schistosomiasis and fascioliasis across host species in Côte d’Ivoire [18–20]. The results will inform the national control program to refine strategies with the ultimate goal of interrupting the transmission of schistosomiasis and STH in northern Côte d’Ivoire.

## Methods

### Ethics statement

Ethical approval was obtained from the National Ethics and Research Committee of Côte d’Ivoire (reference no. 035/MSH/CNER-kp) and the Ethics Committee of North-Western and Central Switzerland (reference no. UBE-2016-00707). District and local authorities, village chiefs, community members, and eligible individuals were informed about the objectives, procedures, potential benefits, and risks associated with the study. Adult volunteers participating in the study signed an informed consent form. A witness was required for adults who were illiterate and children who assented. Participation was voluntary, and hence, participants could withdraw at any time without further obligation.

### Study area and population

**Fig 1:**
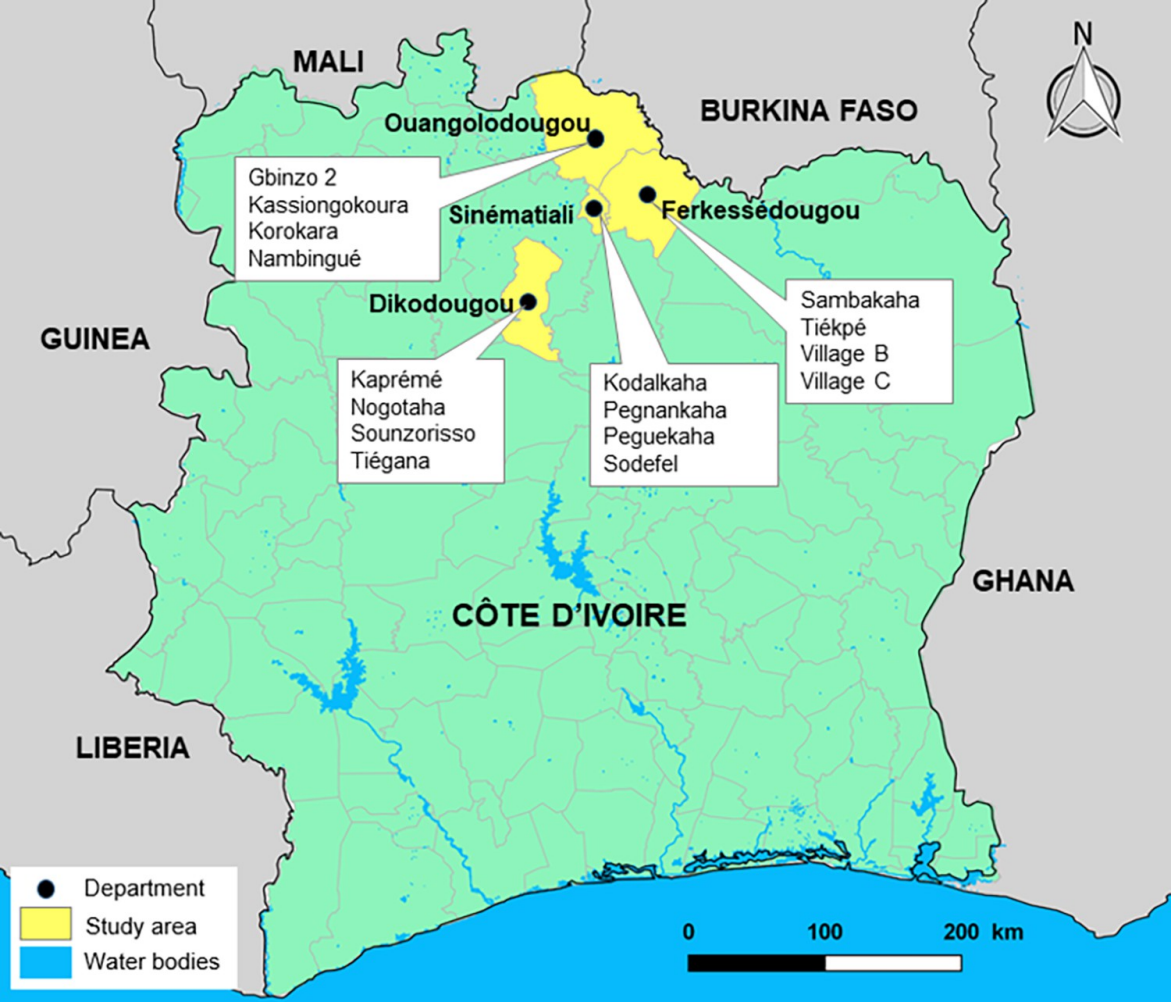
Map of the 16 study sites in the four departments of North Côte d’Ivoire, surveyed for schistosomiasis and soil-transmitted helminthiasis in August 2018. The map was created with QGIS software version 2.16.0 ‘Nødebo’ (QGIS Development Team), using a basemap shapefile from the Database of Global Administrative Areas (GADM, https://gadm.org/; license: https://gadm.org/license.html).

The study was carried out in four departments of North Côte d’Ivoire; namely, Ouangolodougou and Ferkessédougou in the Tchologo region, and Sinématiali and Dikodougou in the Poro region (Fig 1). The North of Côte d’Ivoire is a tropical zone characterized by two seasons: the dry season, which lasts from November to March, and the rainy season from April to October. The mean annual precipitation is approximately 1,000 mm. The hydrologic system is dominated by the Bandama River and its tributaries, and several small multipurpose dams that have been constructed to enhance agricultural production and cattle rearing [21,22]. The population of the four departments was estimated at 477,000 people in 2014 [23].

A total of 16 villages were randomly selected, with four villages in each department. As described in a previous paper [18], these sites were chosen mainly due to the presence of human and animal schistosomiasis and the presence of small dams and water bodies frequented by both humans and livestock. An additional reason was that northern Côte d’Ivoire is the main livestock production area in the country and is an ideal site to investigate the interactions between parasites and their host species, including humans, livestock, and snails. Furthermore, in this region of Côte d’Ivoire, it is believed that elimination can be achieved, and hence, it is important to understand the transmission dynamics amongst all hosts there. People in these villages are mainly from the ethnic group of Sénoufo, but also Peulh, while there are some immigrants from neighboring countries in the North, Burkina Faso and Mali. People in this area are mainly engaged in subsidence farming (e.g., corn, rice, and vegetables). The principal cash crops are cashew nuts, cotton, and mango. Fishing is also common, mainly by non-local people in the man-made lakes.

### Study design and sample size

A cross-sectional survey was carried out in August 2018 to determine the prevalence of human schistosomiasis in the study area. The estimated sample size (*n*) was 553 participants, based on the following formula: *n =* (*Z*^2^ × *p* (1*-p*) × *D*_*eff*_) / *i*^2^, where *Z* = 1.96. The expected prevalence (*p*) based on a previous study is 10% [24], *i* is the precision or margin of the error (5%-points), and *D*_*eff*_ is the design effect (calculated as 1 + (samples per cluster—1) * intracluster correlation coefficient).

### Field procedures

#### Recruitment of study participants

In order to reach the sample size calculated for the departments, 50 participants were recruited per village by convenience and snowball sampling. Participants were recruited after meeting with village authorities and subsequently referred friends and family to participate. Those aged 6 years and above were invited to participate. Written informed consent was obtained at the participants’ homes, after which a questionnaire was administered to collect demographic data (e.g., age, sex, ethnicity, and education attainment), socioeconomic data, and risk factors (e.g., specific water-contact patterns). The field team provided participants with two plastic containers: one for stool and one for urine collection. The next morning, between 8:00 and 12:00 hours, the field team visited the households and collected filled containers from participants. The samples were individually labelled with a unique identification code and transferred to a nearby laboratory for parasitologic examination. In each locality, the field team was assisted by a community health worker, who was familiar with the study villages.

#### Snail sampling

Concurrent to the community survey, a cross-sectional malacologic survey was conducted in waterways of the targeted localities to identify intermediate host snails of *Schistosoma*. Human- and animal-water contact sites were identified with the help of local guides, who were resource persons living in the selected villages. Snail sampling was carried out by two experienced field workers, who searched for and collected snails for 15 min at each site by hand using soft tweezers. In addition, a long-handed kitchen scoop was employed to reach vegetation deeper underwater and to sample collection points that were difficult to access otherwise. Upon collection in the field, the collected snails were identified to genus level and, whenever possible, to species level [25]. The snails were placed in petri dishes, between two layers of cotton wool moistened with site water and wrung out. Snails were placed with the opening against the cotton, without touching. Each dish was labelled with the name and code of the site and the date of collection. The samples were stored in a cooler with a refrigeration device to maintain the temperature between 18 and 23°C.

### Laboratory procedures

#### Parasitologic survey

Stool samples were analyzed using the Kato-Katz technique [26]. In brief, duplicate thick smears were prepared on microscope slides using standard 41.7 mg templates. The slides were allowed to clear for 30–45 min and were then examined under a microscope by two experienced laboratory technicians. The number of eggs of each helminth species was recorded and multiplied by a factor of 24 to obtain the number of eggs per gram (EPG) of stool.

Urine samples were subjected to a filtration method [27]. In brief, 10 ml of urine was vigorously shaken and filtered through a Nytrel filter with a 40 μm mesh size and examined under a microscope for the presence of *S*. *haematobium* eggs, which were counted by experienced laboratory technicians.

#### Cercarial emergence test

Once in the laboratory, the intermediate host snails were exposed to artificial light for 2 hours and observed for cercarial shedding. This process was repeated weekly for one month to allow time for recently infected snails to reach the shedding stage of infection.

### Statistical analysis

Statistical analyses were performed with Stata version 15.0 (Stata Corporation; College Station, TX, United States of America). Participants were stratified into four age groups (6–10, 11–15, 16–20, and >20 years). To account for clustering, the confidence intervals (CIs) of the overall prevalence were adjusted using a constant only, generalised estimating equation (GEE) model with robust sandwich variance estimators. For each participant, the arithmetic mean of the number of species-specific helminth egg counts was calculated based on the duplicate Kato-Katz thick smear readings. At the department level, the geometric mean egg counts were calculated. Helminth infection intensities were classified according to WHO guidelines (Table 1) [28]. Associations between parasitic infections and risk factors (e.g., sex and age) were assessed using GEE analysis for binary outcomes with village as clusters.

**Table 1:** Classification of intensity for schistosome and hookworm infections [28].

Helminth species	Light infection	Moderate infection	Heavy infection
*Schistosoma haematobium*	1–49 eggs per 10 ml of urine	NA	≥50 eggs per 10 ml of urine
*Schistosoma mansoni*	1–99 EPG	100–399 EPG	≥ 400 EPG
Hookworm	1–1,999 EPG	2,000–3,999 EPG	≥4,000 EPG

NA: not applicable; EPG: egg per gram of stool

## Results

### Characteristics of the study population

A total of 742 participants aged 6–96 years from four departments in the northern part of Côte d’Ivoire were included; namely, Ouangolodougou, Ferkessédougou, Sinématiali, and Dikodougou. Males represented 59.0% of the sample, 50.4% of participants were school-age children (5–15 years), and most participants were students, housewives, and crop farmers (Table 2).

**Table 2:** Characteristics of the study population (N = 742) in four departments in North Côte d’Ivoire in August 2018. Numbers in brackets are percentages.

Variable	Ouangolodougou (n = 183)	Ferkessédougou (n = 168)	Sinématiali (n = 193)	Dikodougou (n = 198)	Total
**Sex**					
Male	122 (66.7)	94 (56.0)	112 (58.0)	110 (55.6)	438 (59.0)
Female	61 (33.3)	74 (44.0)	81 (42.0)	88 (44.4)	304 (41.0)
**Age (years)**					
6–10	35 (19.1)	28 (16.7)	57 (29.5)	37 (18.7)	157 (21.2)
11–15	41 (22.4)	58 (34.5)	56 (29.0)	62 (31.3)	217 (29.2)
16–20	15 (8.2)	19 (11.3)	14 (7.3)	19 (9.6)	67 (9.0)
>20	92 (50.3)	63 (37.5)	66 (34.2)	80 (40.4)	301 (40.6)
**Main activity**					
Livestock farmer	24 (13.1)	13 (7.7)	12 (6.22)	15 (7.6)	81 (10.9)
Crop farmer	58 (31.7)	39 (23.2)	37 (19.2)	48 (24.2)	116 (15.6)
Fisherman	0 (0.0)	11 (6.6)	0 (0.0)	0 (0.0)	11 (1.5)
Housewife	51 (27.9)	38 (22.6)	54 (28.0)	49 (24.8)	193 (26.0)
Merchant	2 (1.1)	10 (6.0)	0 (0.0)	3 (1.5)	16 (2.2)
Student	48 (26.2)	57 (33.9)	90 (46.6)	83 (41.9)	278 (37.5)

n, number of participants per department

### *Schistosoma* and hookworm infections

The overall prevalence of schistosomiasis was very low in the study area; 1.9% (95% CI: 0.7–5.3%) and 0.3% (95% CI: 0.1–1.0%) for *S*. *haematobium* and *S*. *mansoni*, respectively (Table 3). At the unit of the department, the prevalence of schistosomiasis was also low; the highest prevalence was observed in Ferkessédougou (5.4%, 95% CI: 2.5–9.9%), followed by Ouangolodougou (2.2%, 95% CI: 0.6–5.5%). At the unit of the village, the prevalence of schistosomiasis was low with one exception; Village C in Ferkessédougou had a moderate prevalence of 21.2% (95% CI: 9.0–38.9%). As regards STH, hookworm was the only species found with a low overall prevalence of 1.5% (95% CI: 0.8–2.8%).

**Table 3:** Prevalence of *Schistosoma haematobium*, *S*. *mansoni*, and hookworm infections in the study population (N = 742) in four departments, North Côte d’Ivoire, August 2018.

Department	n	*S*. *haematobium*		*S*. *mansoni*		Hookworm	
		Infected	% (95% CI)	Infected	% (95% CI)	Infected	% (95% CI)
**Ouangolodougou**	**183**	**4**	**2.2 (0.6–5.5)**	**1**	**0.5 (0.0–3.0)**	**5**	**2.7 (0.9–6.3)**
Gbinzo 2	50	2	4.0 (0.5–13.7)	0	0.0 (0.0–7.1)	1	2.0 (0.0–10.6)
Nambingué	43	1	2.3 (0.1–12.3)	0	0.0 (0.0–8.2)	0	0.0 (0.0–8.2)
Kassiongokoura	46	0	0.0 (0.0–7.7)	0	0.0 (0.0–7.7)	1	2.2 (0.0–11.5)
Korokara	44	1	2.2 (0.1–12.0)	1	2.2 (0.1–12.0)	3	6.8 (1.4–18.7)
**Ferkessédougou**	**168**	**9**	**5.4 (2.5–9.9)**	**0**	**0.0 (0.0–2.2)**	**2**	**1.2 (0.1–4.2)**
Village B	49	2	4.1 (0.5–14.0)	0	0.0 (0.0–7.2)	0	0.0 (0.0–7.2)
Village C	33	7	21.2 (9.0–38.9)	0	0.0 (0.0–10.6)	0	0.0 (0.0–10.6)
Sambakaha	38	0	0.0 (0.0–9.2)	0	0.0 (0.0–9.2)	1	2.6 (0.1–13.8)
Tiépké	48	0	0.0 (0.0–7.4)	0	0.0 (0.0–7.4)	1	2.1 (0.0–11.1)
**Sinématiali**	**193**	**0**	**0.0 (0.0–1.9)**	**1**	**0.5 (0.0–2.9)**	**0**	**0.0 (0.0–1.9)**
Sodefel	49	0	0.0 (0.0–7.2)	1	2.0 (0.0–10.8)	0	0.0 (0.0–7.2)
Peguékaha	49	0	0.0 (0.0–7.2)	0	0.0 (0.0–7.2)	0	0.0 (0.0–7.2)
Pegnankaha	50	0	0.0 (0.0–7.1)	0	0.0 (0.0–7.1)	1	2.0 (0.0–10.6)
Kodalkaha	45	0	0.0 (0.0–7.9)	0	0.0 (0.0–7.9)	0	0.0 (0.0–7.9)
**Dikodougou**	**198**	**0**	**0.0 (0.0–1.8)**	**0**	**0.0 (0.0–1.8)**	**4**	**2.0 (0.6–5.1)**
Tiégana	49	0	0.0 (0.0–7.2)	0	0.0 (0.0–7.2)	1	2.0 (0.0–10.8)
Kaprémé	50	0	0.0 (0.0–7.1)	0	0.0 (0.0–7.1)	2	4.0 (0.5–13.7)
Nogotaha	50	0	0.0 (0.0–7.1)	0	0.0 (0.0–7.1)	0	0.0 (0.0–7.1)
Sounzorisso	49	0	0.0 (0.0–7.2)	0	0.0 (0.0–7.2)	1	2.0 (0.0–10.8)
**Total**	**742**	**14**	**1.9 (0.7–5.3)** ^**§**^	**2**	**0.3 (0.1–1.0)** ^**§**^	**11**	**1.5 (0.8–2.8)** ^**§**^

n, number of participants per department or village; CI, confidence interval

^**§**^, confidence intervals adjusted to account for clustering with GEE model

**Fig 2:**
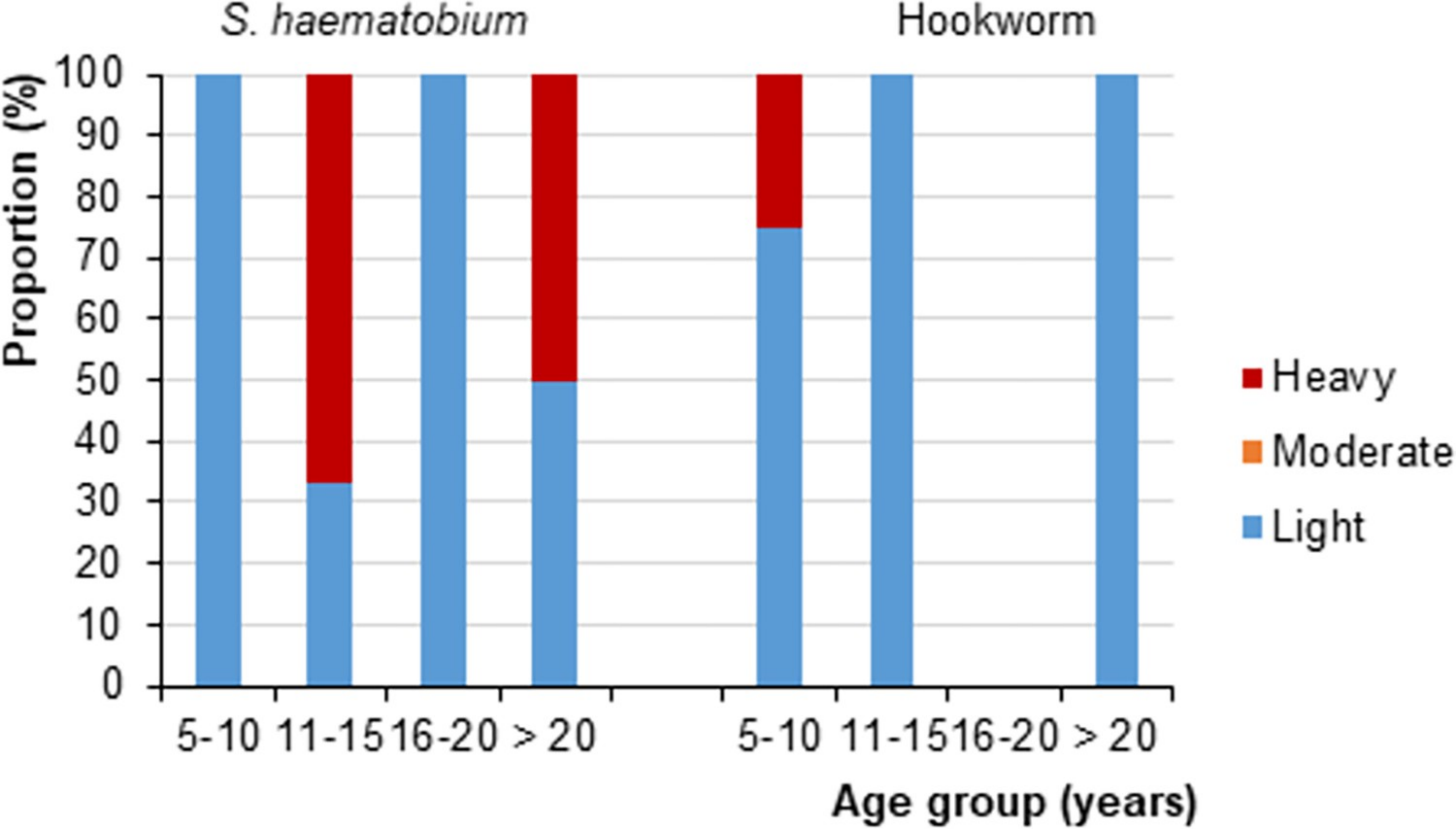
Intensity categories of *S*. *haematobium* and hookworm infection, stratified by participant age groups, in four departments of North Côte d’Ivoire in August 2018. For the definition of infection intensities, see Table 1.

Low average infection intensities of the two schistosome species and hookworm were observed in all four departments with geometric means of 16.2 eggs per 10 ml of urine (95% CI: 4.2–62.7 per 10 ml of urine) for *S*. *haematobium*, 93.0 EPG (95% CI: 0.4–24,198 EPG) for *S*. *mansoni*, and 156.5 EPG (95% CI: 52.1–470.3 EPG) for hookworm. Infection intensities were also generally low for all three parasites with over half of those infected having low infection intensities (Fig 2). However, high infection intensities were observed in those aged 11–15 years (67%) and older than 20 years (50%) for *S*. *haematobium*, and in 5- to 10-year-old children (25%) for hookworm (Fig 2).

**Fig 3:**
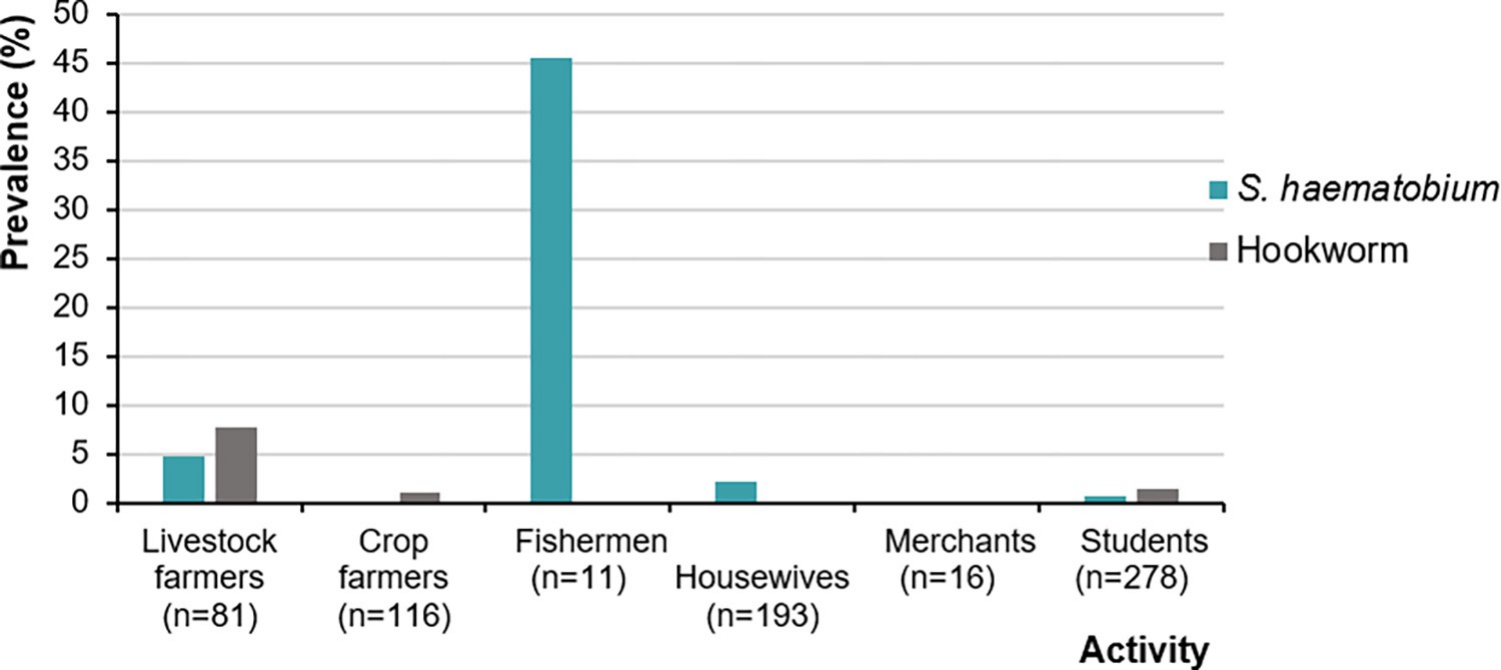
Prevalence of *Schistosoma haematobium* and hookworm, stratified by occupation, in four departments of North Côte d’Ivoire in August 2018.

Table 4 shows the association between an infection (*S*. *haematobium* and hookworm), and sex or age. *S*. *mansoni* infection was not considered in the analysis, because of the very low prevalence (2/742). Males had a higher risk of infection with *S*. *haematobium* compared to females, but the difference was not statistically significant (2.3% versus 1.3%, odds ratio [OR]: 1.5, 95% CI: 0.8–2.7). Males had higher odds of hookworm infection than females, but the difference lacked statistical significance (2.3% versus 0.3%, OR: 5.5, 95% CI: 0.6–53.7). Participants aged 16–20 years had higher odds of infection with *S*. *haematobium* than other age groups (OR: 5.3, 95% CI: 1.8–15.7), and fishermen had the highest odds of *S*. *haematobium* infection (Fig 3).

**Table 4:** Association of infection (*Schistosoma haematobium* and hookworm) with sex and age among 742 participants from four departments in North Côte d’Ivoire in August 2018.

Factors	n	*Schistosoma haematobium*				Hookworm			
		Infected (%)	OR	95% CI^λ^	p-value	Infected (%)	OR	95% CI^λ^	p-value
**Sex**									
Female	304	4 (1.3)	1.0	-	-	1 (0.3)	1.0	-	-
Male	438	10 (2.3)	1.5	0.8–2.7	0.16	10 (2.3)	5.5	0.6–53.7	0.15
**Age (years)**									
5–10	157	2 (1.3)	1.0	-	-	4 (2.6)	1.0	-	-
11–15	217	6 (2.8)	2.3	0.7–7.5	0.19	5 (2.3)	1.0	0.3–3.3	0.95
16–20	67	4 (6.0)	5.3	1.8–15.5	0.003	0 (0.0)	-	-	-
>20	301	2 (0.7)	0.6	0.2–2.0	0.40	2 (0.7)	0.4	0.1–2.8	0.34

n, number of participants; % percentage of infected people

^λ^ Confidence intervals (CI) adjusted to account for clustering

### Results of malacologic surveys

A total of 111 human- and animal-water contact sites were identified at 47 water sources in the study area. The number of these contact sites ranged between 18 (Dikodougou) and 35 (Ferkessédougou). These contact sites were distributed amongst seven water sources in Sinématiali, 11 in Dikodougou, 11 in Ouangolodougou, and 18 in Ferkessédougou. Small dams (n = 22) were the most common human- or animal-water contact sites, followed by rivers (n = 13) and ponds (n = 12).

A total of 1,273 freshwater snails belonging to seven genera were collected, namely, *Biomphalaria*, *Bulinus*, *Gabbiella*, *Gyraulus*, *Lanistes*, *Lymnaea*, and *Melanoides* (Table 5). *Bulinus forskalii* (n = 761) was the most abundant snail species in the study area, consisting of over half of the snails collected. Concerning schistosomiasis, potential intermediate host snails were *Bu. forskalii* (NB: bring on one line; “forskalii” not capital) (n = 761), *Bulinus truncatus* (n = 205), and *Biomphalaria pfeifferi* (n = 1). From these snails, one specimen of *Bu*. *truncatus* was found to be shedding schistosome cercariae, while some snails were observed shedding several zoophilic cercariae not pathogenic to humans, namely, strigeids, echinostomes, and xiphidiocercariae (Table 6).

**Table 5:** Snail species identified in 16 localities from four departments in North Côte d’Ivoire in August 2018.

Locality	*Biomphalaria pfeifferi* ^δ^	*Bulinus forskalii* ^δ^	*Bulinus truncatus* ^δ^	*Gabbiella africana*	*Gyraulus* sp.	*Lanistes ovum*	*Lymnaea natalensis*	*Melanoides tuberculata*	Total
**Ouangolodougou**	**0**	**153**	**98**	**53**	**0**	**7**	**7**	**0**	**318**
Gbinzo 2	0	82	86	0	0	0	0	0	168
Nambingue	0	37	0	52	0	0	0	0	89
Kassiongokoura	0	7	0	1	0	0	0	0	8
Korokara	0	27	12	0	0	7	7	0	53
**Ferkessédougou**	**1**	**257**	**82**	**17**	**5**	**13**	**8**	**68**	**451**
Sodesucre B	0	3	0	0	0	0	0	0	3
Sodesucre C	1	207	0	17	5	13	7	68	318
Sambakaha	0	38	82	0	0	0	1	0	121
Tiépké	0	9	0	0	0	0	0	0	9
**Sinématiali**	**0**	**305**	**20**	**50**	**0**	0	**1**	**0**	**376**
Sodefel	0	0	0	0	0	0	0	0	0
Peguékaha	0	305	20	40	0	0	0	0	365
Pegnankaha	0	0	0	10	0	0	1	0	11
Kodalkaha	0	0	0	0	0	0	0	0	0
**Dikodougou**	**0**	**46**	**5**	**34**	**1**	**15**	**27**	**0**	**128**
Tiegana	0	1	5	34	0	8	25	0	73
Kapreme	0	0	0	0	0	0	0	0	0
Nogotaha	0	0	0	0	0	0	0	0	0
Sounzorisso	0	45	0	0	1	7	2	0	55
**Total**	**1**	**761**	**205**	**154**	**6**	**35**	**43**	**68**	**1,273**

^δ^ Schistosome intermediate host snails

**Table 6:** Intermediate host snails of schistosomes shedding cercariae in 16 localities from four departments in North Côte d’Ivoire, August 2018.

Snail species	n	Cercariae (%)				
		Schistosomes	Echinostomes	Strigeides	Xiphidio-cercariae	Total
***Biomphalaria pfeifferi***	1	0 (0.0)	0 (0.0)	0 (0.0)	0 (0.0)	0 (0.0)
***Bulinus forskalii***	761	0 (0.0)	17 (2.2)	0 (0.0)	2 (0.3)	19 (2.5)
***Bulinus truncatus***	205	1 (0.5)	0 (0.0)	1 (0.5)	1 (0.5)	3 (1.5)
**Total**	967	1 (0.1)	17 (1.8)	1 (0.1)	3 (0.3)	22 (2.3)

n, number of individuals per snail species

## Discussion

In the new WHO road map, schistosomiasis and STH are targeted for elimination as a public health problem by 2030 [7]. The prevalence and risk factors of schistosomiasis and STH were assessed in four departments in the northern part of Côte d’Ivoire. Overall, a very low prevalence of *S*. *mansoni* (0.3%) and a somewhat higher, but still very low, prevalence of *S*. *haematobium* (1.9%) were found. Hookworm was the only STH observed with a very low overall prevalence (1.5%), and infection intensities were mainly low.

The observations in North Côte d’Ivoire confirm the low endemicity of schistosomiasis. Indeed, this part of Côte d’Ivoire is known to have low to moderate prevalence of schistosomiasis [16]. In 2015, a study in Korhogo noted a low prevalence of 1.9% for both urogenital and intestinal schistosomiasis among school-age children [29]. The low prevalence of schistosomiasis and STH might be explained by climatic factors and large-scale drug administration using praziquantel against schistosomiasis and albendazole against STH, which commenced in 2013 [16,17]. Of note, the increasing supply of safe drinking water in rural areas after a decade-long socio-political crisis (2002–2011) has contributed to the reduction of the population’s contact with cercarial-infested freshwater. Nevertheless, at the unit of the village, there was one study site in the department of Ferkessédougou that showed a moderate prevalence of *S*. *haematobium* (21.2%), and hence, is eligible for preventive chemotherapy with praziquantel [2]. This level of moderate endemicity is likely related to a small multipurpose dam in close proximity to the village, with which the population is in frequent contact for farming or cattle watering. Dams have long been known to be associated with an elevated risk of schistosomiasis [30–32]. Although the prevalence of schistosomiasis is low in North Côte d’Ivoire, it is worth mentioning that livestock schistosomiasis is widespread in this part of the country [18] and hybridization between *S*. *bovis* (cattle schistosome) and *S*. *haematobium* has recently been observed in humans [10,12]. The use of new and more sensitive techniques, such as molecular tools, is needed in this low-endemicity context in order to detect light-intensity infections that evade detection with the standard Kato-Katz technique [33]. When evaluating the prevalence of schistosomiasis, preschool-age children (< 6 years) that were not included in this study, should be considered in order to have a complete overview of the situation. In fact, preschool-age children may be infected when accompanying their caregivers to water bodies that are likely to be infested with schistosome cercariae [34,35]. The new formulations of praziquantel for preschool-age children is a welcome addition to the armamentarium for reducing the burden of schistosomiasis in this age group, which has not been targeted for a long time by mass drug administration campaigns [36].

The somewhat higher prevalence of *S*. *haematobium* compared to *S*. *mansoni* was also observed in previous studies [16,37]. This observation could be explained by the distribution of intermediate host snails. In fact, *Bu*. *truncatus* and *Bu*. *globosus*, two important intermediate host snails of *S*. *haematobium*, seem to be more abundant in northern Côte d’Ivoire than *Bi*. *pfeifferi*, which serves as the intermediate host snail of *S*. *mansoni* in Côte d’Ivoire. Previous research revealed that *S*. *mansoni* is the predominant schistosome species present in the West and South of Côte d’Ivoire [35,38,39].

Concerning risk factors, both males and females were exposed to *S*. *haematobium* infection. Adolescents and young adults (age range 16–20 years) were at a higher odds of being infected with *S*. *haematobium* than their younger or older counterparts. In fact, this age group might include males who fish, a high risk activity for schistosome infection because it involves frequent and prolonged contact with potentially infested freshwater sources [13,40,41]. It should be noted that high intensities of *S*. *haematobium* infection have been observed in children aged 11–15 years, reinforcing that this age group is at risk for schistosomiasis. School-age children are reported to engage in activities such as bathing and swimming in open water [13]. Furthermore, it is common practice in the study area for males aged 11–15 years to drive cattle from pasture to water at rivers and small dams. This practice frequently exposes young males to cercarial-infested water sources.

Males were at a higher odds of hookworm infection than females (OR: 5.5, 95% CI: 0.6–53.7). In our study, males were exposed to hookworm infection through activities such as farming and animal husbandry. Indeed, open defecation, a major risk factor for hookworm infection [42], is practiced on the outskirts of villages in areas that are ploughed and used for maize cultivation by adult males during the rainy season. The maize areas are abandoned and occupied by weeds in the dry season, during which time open defecation is practiced. A study in the departments of Taabo, Toumodi, and Djékanou in the south-central part of Côte d’Ivoire found that males were at a higher odds of hookworm infection than females due to the high level of open defecation on rural cocoa farms [43].

The malacologic survey confirmed the presence of *Bi*. *pfeifferi*, *Bu*. *truncatus*, and *Bu*. *forskalii* in North Côte d’Ivoire, corroborating previous findings [12,44]. However, only one specimen (0.5%) of *Bu*. *truncatus* was found to be shedding schistosome cercariae. This low infection rate in intermediate host snails is in line with the observed low prevalence of human schistosomiasis. It is not a rare phenomenon to observe a very low prevalence of schistosome cercariae shedding in natural areas. An absence of snails shedding schistosome cercariae was reported from a 3-year study assessing the dynamics of freshwater snails and schistosomiasis prevalence in school children during the construction and operation of a multipurpose dam in the central part of Côte d’Ivoire [45]. Similar results were observed from a study on characteristics of persistent hotspots of *S*. *mansoni* in western Côte d’Ivoire [46]. In contrast, a study from a Schistosomiasis Consortium for Operational Research and Evaluation (SCORE) project assessing the molecular characterization and distribution of *Schistosoma* cercariae collected from naturally infected bulinid snails in northern and central Côte d’Ivoire reported that only 1% (25/2,417) of both *Bu*. *truncatus* and *Bu*. *globosus* were shedding schistosome cercariae [12]. That study also showed that the snails are abundant in the dry season, but the proportion of snails shedding cercariae remains quite low in all seasons [12].

This study has several limitations that are offered for discussion. First, participants were not randomly selected; the convenience and snowball sampling method used may have selected for health conscientious individuals, leading to an underestimation of the reported infection prevalence. At the time of sampling many inhabitants were away from the villages, which significantly limited the number of people available to participate. This limits the generalizability of the results. Secondly, only single stool and urine samples were collected, and hence, infection prevalence of schistosomes and hookworm are likely to be underestimated, particularly in this low endemicity setting [47].

## Conclusions

Prevalence and transmission of schistosomiasis and hookworm in the northern part of Côte d’Ivoire is very low. This calls for attempts to interrupt transmission, as has been tried in a large cluster-randomized trial over a 3-year period prior to this study [48]. Although *S*. *haematobium* transmission could not be fully interrupted, preventive chemotherapy, coupled with mollusciding, resulted in marked prevalence reduction. Longer term efforts, combining preventive chemotherapy with snail control, information, education, and communication (IEC), and improved sanitation are warranted so that the ultimate goal of elimination of schistosomiasis and hookworm in these seasonal transmission settings might be achieved.
